# Club Drugs and Psychiatric Sequelae: An Issue of Vulnerability and Previous Psychiatric History

**DOI:** 10.3390/ijerph18136944

**Published:** 2021-06-29

**Authors:** Giovanni Martinotti, Cristina Merino Del Villar, Andrés Garcia Cordoba, Lluís Andrés Tubau, Ivan Castro Sánchez, Francesco Di Carlo, Stefania Chiappini, Mauro Pettorruso, Fabrizio Schifano, Massimo Di Giannantonio

**Affiliations:** 1 Department of Neuroscience, Imaging, Clinical Sciences, University G. d’Annunzio, 66100 Chieti-Pescara, Italy; francesco.dic@hotmail.it (F.D.C.); stefaniachiappini9@gmail.com (S.C.); mauro.pettorruso@hotmail.it (M.P.); digiannantonio@unich.it (M.D.G.); 2 Psychopharmacology, Drug Misuse and Novel Psychoactive Substances Research Unit, School of Life and Medical Sciences, University of Hertfordshire, Hertfordshire AL10 9EU, UK; f.schifano@herts.ac.uk; 3 Can Misses Hospital, Carrer de Corona, s/n, 07800 Eivissa, Illes Balears, Spain; cmerino@asef.es; 4 Emergency Staff Group, Calle Pere Francés 43, 07800 Eivissa, Illes Balears, Spain; andres@emergencystaff.es (A.G.C.); eleonorachillemi@hotmail.it (L.A.T.); ivcastro@yahoo.es (I.C.S.); 5 Plan Municipal Sobre Drogas, Ayuntamiento de Ibiza, Carrer de Canàries, 35-Edif. CETIS, 1r piso, 07800 Eivissa, Illes Balears, Spain

**Keywords:** club drugs, psychopathological consequences, psychosis, substance use disorder

## Abstract

The pursuit of pleasure among clubbers and disco-goers often involves drug use. However, whether substance use may represent a relevant risk factor contributing to the development of psychiatric symptoms and of mental illness remains debated. The purposes of this study were to evaluate the percentage of subjects who developed long-lasting psychiatric symptoms in a sample of subjects reporting use of substances in nightclubs, and to evaluate the role of a previous psychiatric diagnosis in these subjects. Data were collected during three consecutive years in dedicated nursing units inside all the nightclubs of Ibiza, in emergency hospital rooms at the Can Misses Hospital and inside the psychiatric ward. A total of 10,163 subjects required medical assistance inside discos in the medical-nursing units, of which 223 required transfers to hospital emergency rooms. Of these, 110 required subsequent psychiatric hospitalization. Ninety-one (82.7%) of these patients had a positive psychiatric history, which was also found in thirty-one of the 113 subjects (27.4%) not requiring psychiatric hospitalization. Negative psychiatric history was negatively associated with hospitalization (Coefficient = −2.574; *p* = 0.000) and for subjects with a negative psychiatric history the odds to be hospitalized changed by a factor of 0.076. Gender, age, civil status and nationality were not significant predictors of hospitalization. Overall, the number of subjects who developed major psychiatric disorders appeared to be limited. However, the presence of a psychiatric history here played a crucial role. Club drugs are therefore able to induce psychiatric sequelae requiring hospitalization mainly in subjects who are already vulnerable from a psychopathological point of view.

## 1. Introduction

The use of psychoactive substances, although extemporized, unplanned and limited in time, can have a variety of consequences on health, including the onset of serious psychopathological symptoms eventually requiring hospitalization. Therefore, timely health policies and consistently updated responses by health professionals are needed to promote harm reduction [1].

In recent years, the global situation concerning occasional use and substance use disorders (SUD) in general has undergone a rapid, but not always positive, evolution. The market entry of readily available new psychoactive substances (NPS), the presence of traditional ‘enhanced’ substances and the phenomenon of ‘nightlife’ or ‘clubbing’ associated with international travels have dramatically complicated the scenario, including in relation to possible management and treatment strategies of drug use. Holiday periods, particularly in summer, appear to represent a time of risk, excess, and experimentation, especially among young people [2]. In these contexts, hedonistic partying is socially accepted, and drugs are typically readily available. Polysubstance use (i.e., the consumption of two or more compounds simultaneously) [3,4], chemsex and other behaviors are frequently reported, and such activities involve a variety of traditional drugs and NPS, such as methamphetamines, synthetic cathinones, synthetic cannabinoids, opioids, naturally derived drugs and prescription drugs [5,6,7]. However, data regarding the number of ‘clubbers’ and party-goers reporting moderate to severe psychiatric symptoms and requiring intensive inpatient treatment remain unclear and not adequately studied in the literature.

Ibiza has for many years been considered the leading island in the field of entertainment and music, with a growing number of visitors looking for ‘highs’, opportunities for transgression and psychoactive substances. This has been transforming the island into a sort of open-air laboratory for the experimentation of new and powerful psychoactive substances provided by drug dealers, with an increased number of aggression episodes, induced psychiatric syndromes and fatalities [8,9].

Several studies have shown how the use of certain substances, even extemporaneously, can cause the development of possible psychopathological symptoms that are not always reversible, as in the case of cannabis-induced psychosis. Recently, researchers have reported that the use of both traditional ‘enhanced’ drugs and NPS may be associated with the onset of a variety of psychiatric symptoms and conditions, including changes in consciousness; auditory and visual hallucinations; paranoid thoughts, such as delusions of reference, persecution, grandeur and jealousy; dissociation; confusion; insomnia; chronic cognitive impairment; hypomanic states; aggression; violence; and suicidal thoughts [10,11,12]. These symptoms are often due to the greater potency of the substances currently available on the market compared to older generations of substances, as well as their action on a number of different neural pathways, including dopamine and serotonin receptors for psychedelic phenethylamines, tryptamines and cathinones, cannabinoid receptors for synthetic cannabinoids and N-methyl-D-aspartate (NMDA) receptors for dissociative anesthetic drugs and others [13].

It is of great interest to understand the extent to which the use of substances in subjects not seeking treatment and in an apparent state of well-being can lead to the development of psychopathological symptoms of such severity as to determine a hospitalization in psychiatry. It is also of fundamental importance to understand the role of a possible previous psychiatric history in relation to the development of induced reactions of psychopathological interest, with variable consequences over the time horizon and with different evolutions in terms of outcome.

The aim of the present study was (a) to analyze the number of subjects reporting a psychiatric condition requiring hospitalization in a psychiatric inpatient unit after the use of substances in a nightclub scenario, and (b) to report the role of a previous psychiatric antecedent in relation to the treatment outcome.

## 2. Materials and Methods

In this study, we collected all the cases of subjects who, following the intake of psychoactive substances in a disco, reported psychopathological symptoms during the summer opening periods between May 2015 and October 2018, (from the second weekend of May for ‘opening’ parties to the second weekend of October for ‘closing’ parties). The study was conducted in three phases.

In phase 1, treatment-seeking subjects admitted to the emergency staff nursing units inside all the nightclubs of Ibiza were recruited for the study. Inclusion criteria were represented by (1) the presence of alcohol or substance intoxication (mild, moderate or severe) and (2) the presence of psychiatric symptoms as evaluated by a trained psychiatric nurse.

In phase 2, a subsample of subjects was transferred to the Can Misses Hospital emergency rooms for medical evaluation. Patients with delirium tremens, epilepsy, liver encephalopathy, dementia and other neurological diseases, severe cardiac failure, diabetes mellitus, severe liver impairment, kidney failure or neoplastic diseases were excluded after clinical evaluation. Psychiatric history was evaluated through a standard clinical interview based on DSM-5 criteria. In order to confirm the use of substances, a urine sample was collected upon admission, stored at −30 °C and subsequently analyzed at the laboratory of the Department of Forensic Toxicology of the Marche Polytechnic University in Ancona, Italy. Gas chromatography/mass spectrometry was used to confirm the consumption of psychoactive substances and/or prescription drugs.

In phase 3, a further subsample of subjects was admitted to the psychiatric ward of Can Misses Hospital. All patients were evaluated according to the DSM-5 diagnostic classification. Demographic (i.e., age, gender, family, and nationality) and socioeconomic data (i.e., living status, job status, and level of education) were collected in a structured interview administered during hospitalization after the resolution of intoxication symptoms. The interview investigated each patient’s recent and past medical and psychiatric history in addition to alcohol and substance use habits (including tobacco, caffeine, cannabis, cocaine, heroin, and novel psychoactive substances). Detailed data were reported elsewhere [14].

### 2.1. Ethics

Data collection was carried out in an anonymous and confidential way; all participants received a detailed explanation of the design of the study, and written informed consent was systematically obtained from every subject according to the Declaration of Helsinki. Ethics approval was granted by the University of Hertfordshire Health and Human Sciences ECDA, protocol no. aPHAEC1042 (03); by the CEI Illes Balears, protocol no. IB 2561/15 PI; and by the University ‘G. d’Annunzio’ of Chieti-Pescara, no. 7/09 04-2015. A Majorcan local ethics committee also gave approval to the study.

### 2.2. Data Analysis

Statistical analysis was performed using IBM SPSS® Statistics software, version 20.0 (IBM Corp, Armonk, NY, USA) Binary logistic regression was performed to evaluate whether there was a significant association between the dependent categorical variable admission to the psychiatric unit (hospitalization YES, coded as 1; NO, coded as 0) and the independent categorical variables: psychiatric history (POS = positive, coded as 0, NEG = negative, coded as 1), gender (M = male, coded as 0, F = Female, coded as 1), nationality (E= European, coded as 1, NE = Other, coded as 0), civil status (1) (SNM = single/never married coded as 0, M = married coded as 0, D= divorced, coded as 1), (2) (SNM = single/never married coded as 0, M = married coded as 1, D= divorced, coded as 0), as well as the independent continuous variable age. A *p*-value of <0.05 was considered statistically significant.

## 3. Results

**Figure 1:**
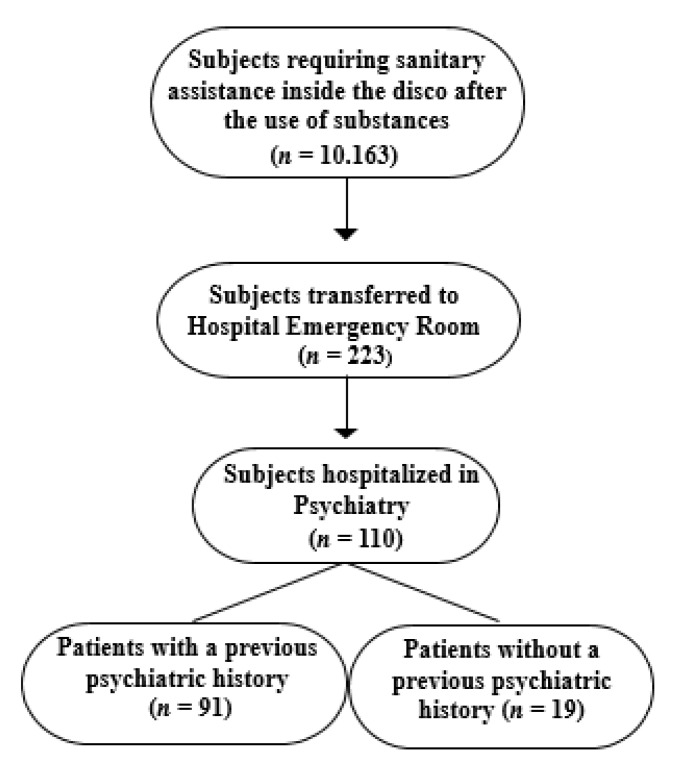
Diagram of subject flow.

A total of 10,163 subjects required sanitary assistance inside the discos in a dedicated area; of these, 223 required to be transferred to the Can Misses Hospital emergency room for support and treatment, with some even in conditions of intoxication (70; 31.4%), with disturbances in the level of consciousness, cognition, perception, judgement, affect, or behavior and in need of assisted ventilation during transport. Of the subjects who entered the emergency room, 110 (49.3%; M/F: 2.2; age: 32.6 ± 9.2) required a subsequent hospitalization in psychiatry, whereas 113 (50.7%) needed a 2 to 36 hour stay in the emergency department before a rapid home discharge. The sociodemographic characteristics of subjects with or without a subsequent hospitalization are shown in Table 1 and subject flow is described in Figure 1. The group of subjects requiring a subsequent hospitalization in psychiatry was characterized by a high level of employment (47.3%), and a living status mostly characterized by living with parents (26.4%) and alone (24.5%). Ninety-one (82.7%) patients requiring hospitalization had a positive psychiatric history, which was also found in thirty-one of the 113 subjects (27.4%) not requiring psychiatric hospitalization. The logistic regression model revealed a negative association between negative psychiatric history and hospitalization (coefficient = −2.574, std. error = 0.348, Wald = 54.656, df = 1, *p* = 0.000, 95% CI = 0.0385 to 0.1509) and that subjects with a negative psychiatric history were less likely to be hospitalized (odds ratio = 0.076), whereas gender, age, civil status and nationality were not significant predictors (*p* = 0.05), suggesting that previous psychiatric history played a crucial role in hospitalization due to the psychiatric consequences after the use of substances (subject flow is described in Figure 1). 

The most common psychiatric antecedents were represented by the following diagnoses: SUD, bipolar disorder, schizophrenia spectrum disorder and borderline personality disorder. Psychodisleptics (45%; mainly cannabis and empathogens) followed by psychostimulants (40%; mainly cocaine and methamphetamine) were the classes of drugs mainly prevalent in urine samples, with almost half of the participants (46%) declaring to have used a substance without knowing what it was. The average stay in the psychiatric ward was 23 days, and the obligation to carry out restraints was reported in 14 cases (4%). Finally, psychiatric diagnoses of hospitalized patients recorded were: Cluster B personality disorder (*n* = 34), schizophrenia spectrum disorder (*n* = 33), mood disorder (*n* = 22), substance use disorder (*n* = 44), alcohol use disorders (*n* = 16), and anxiety disorder (*n* = 3).

## 4. Discussion

**Figure 2:**
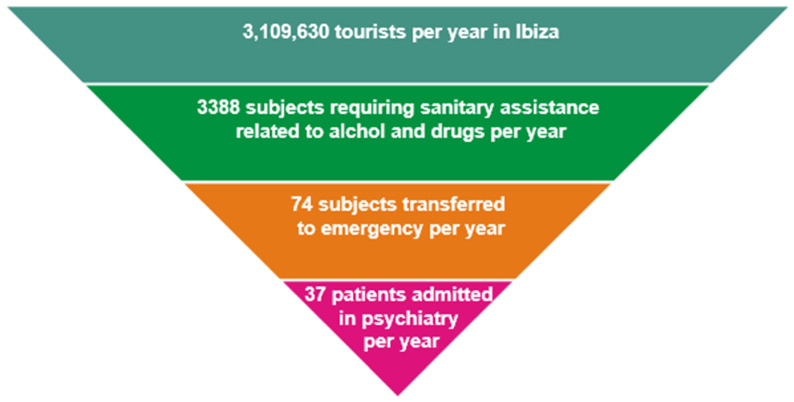
Tourists per year in Ibiza versus number of subjects requiring different typologies of sanitary assistance for drug-induced psychopathological consequences.

In this study performed during three consecutive tourist seasons, we reported a low number of subjects who reported psychopathological consequences after the use of substances, such as the requirement of hospitalization in psychiatry. Considering that the number of tourists in Ibiza reaches an average of 3,109,630 per year with a prevalent number of clubbers among them [15,16] we believe the number of subjects developing a psychiatric hospitalization to be limited (Figure 2) despite the spread of substances that are potent from a pharmacodynamic point of view with documented psychotomimetic properties [17]. 

The low incidence of psychiatric consequences could have been determined by the high resilience of this specific sample of subjects. Festival-goers and club-goers in holiday resorts—environments in which hedonistic partying is socially accepted and drugs are typically readily available—might be less prone to developing psychiatric consequences in reason of a low prevalence of previous psychiatric antecedents. The characteristics of our sample, with high levels of education and good employment rates, differ from the typical profile of substance abusers, who are frequently characterized by unemployment, low levels of education, low quality of life, precarious housing conditions, low socioeconomic level and the presence of psychiatric comorbidities [18,19,20]. An explanation for this phenomenon might be that the characteristics of substance-using clients have changed over recent years, with recreational drug users differing greatly from the ‘drug addicts’ of the past [21,22]. Drug abuse used to represent a way to escape from social norms, whereas nowadays is largely integrated among cultural post-modern rituals, specifically in groups of adolescents and young adults [23]. The drug user in the club and disco scenario is often highly integrated in the social context, of a high socioeconomic bracket, with a good level of education and placed in an adequate peer group able to provide support. These factors can contribute to making the subject more resilient and able to withstand, at least in the short term, the possible psychopathological consequences derived from the use of certain substances. 

It should be noted that in our sample, the use of substances resulted in many cases of multiple use, sometimes unknown to the user. Previous research has established that individuals with substance use disorder and co-occurring mental illness are more likely to engage in high-risk behaviors, such as polysubstance use [24]. Our study shows that polyabuse is instead a typical behavior, regardless of the presence of comorbidities, and likely represents a typical typology of use in relation to the context and its socio-cultural aspects [23].

The second major point that emerged from this study is the role of psychiatric history, which played a crucial role in the subsequent development of long-term psychiatric consequences. This finding is consistent with those of other studies on subjects with dual disorders, with higher levels of psychiatric symptoms being reported in subjects with a pre-existing psychiatric history exacerbated by the use of substances [25,26]. Co-occurring psychiatric disorders, and specifically mood and anxiety disorders, appear to be prevalent in vulnerable populations of substance users [27]. In the context of methamphetamine use, symptoms of anxiety and panic are likely to be further exacerbated by the physiological effects of stimulant intoxication, whereas a family history of a primary psychotic disorder and comorbid psychiatric symptoms remained significantly associated with persistent psychotic symptoms after adjusting for the severity of methamphetamine dependence [28].

This study presents several limitations: (1) the assessment of psychiatric antecedents was based on a psychiatric assessment without a structured interview; (2) the sample of subjects evaluated inside the disco for sanitary assessment and not requiring further hospitalization was not evaluated by psychiatrists but by trained nurses; (3) a few subjects requiring hospitalization could have benefited from health services and subsequent private hospitalization, thus circumventing the proposed count; (4) the long-term effects of novel and traditional substances were not adequately evaluated, as well as a deep analysis of the previous use of substances; and (5) an accurate urinalysis and medication history of patients who did not require subsequent admission to psychiatry was not possible in the emergency setting, thus not allowing adequate comparison with the group of patients subsequently admitted to psychiatry.

In future studies, the following points shall be addressed: (1) better discrimination of the psychopathological effects of specific substances, including NPS, in relation to dosages and route of assumption; and (2) a prospective look at the long-term effects of substances.

## 5. Conclusions

In our sample of clubbers and disco-goers, the role of a positive psychiatric history was found to be a crucial factor for determining a subsequent hospitalization after an episode of substance misuse, with cases of high levels of psychiatric symptoms also requiring restraints. Novel and vintage psychoactive substances are therefore able to determine long-term psychiatric sequalae mainly in subjects already previously compromised and vulnerable from a psychopathological point of view. Therefore, mental health assessments should routinely be performed in an emergency setting and in the early stages of treatment [27]. Due to these data, harm reduction strategies and prevention campaigns dedicated to subjects with positive psychiatric histories could represent a valuable option. At the same time, and also in light of the relatively low percentage of subjects requiring an impatient admission to psychiatric unit, this study suggests that a strong emphasis on prohibitionist models of intervention could not be of clinical impact. Prospective studies are warranted in order to understand the long-term impact of substance use in club scenarios.

## Figures and Tables

**Table 1:** Sociodemographic characteristics of subjects with or without a subsequent hospitalization.

Characteristic	*n* (Requiring Hospitalization)	%	*n* (Not Requiring Hospitalization)	%
**SAMPLE SIZ**E	110		113	
**Gender**				
Male	76	69.1	73	64.6
Female	34	30.9	40	35.4
**Nationality**				
European	79	71.8	82	72.6
Other	30	27.3	31	27.4
Not available (NA)	1	0.9	0	0
**Civil status**				
Single/never married	66	60	67	59.3
Divorced	21	19.1	23	20.4
Married	13	11.8	23	20.4
NA	10	9.1	0	0
**Category of substances**				
Psychodysleptics	49	44.5	NA	-
Psychostimulants	44	40	NA	-
Psychodepressors	17	15.5	NA	-
**Groups of substances**				
Stimulants	74	67.3	NA	-
Cannabinoids	68	61.8	NA	-
Depressors	32	29.1	NA	-
Empathogens	28	25.5	NA	-
Dissociatives	15	13.6	NA	-
Opioids	9	8.2	NA	-
Psychedelics	7	6.4	NA	-
	Range (years)	Mean (SD)	Range (years)	Mean (SD)
**Age**	19–63	32.57 (9.2)	19–63	32.33 (9.3)
**Total years of education**	5–27	12.91 (4.5)	NA	-
**Age subgroups**				
**≤30 yo**	57	51.8	55	48.7
**31–50 yo**	47	42.7	52	46
**>50 yo**	6	5.5	6	5.3
